# Ketone production by ketogenic diet and by intermittent fasting has different effects on the gut microbiota and disease progression in an Alzheimer’s disease rat model

**DOI:** 10.3164/jcbn.19-87

**Published:** 2020-03-20

**Authors:** Sunmin Park, Ting Zhang, Xuangao Wu, Jing Yi Qiu

**Affiliations:** 1 Department of Food and Nutrition, Obesity/Diabetes Research Center, Hoseo University, 165 Sechul-Ri Baebang-Yup, Asan-si, Chungchungnam-Do, 336795, Korea

**Keywords:** ketone, amyloid-β, hippocampus, insulin signaling, gut microbiome

## Abstract

The benefits of ketone production regimens remain controversial. Here, we hypothesized that the ketone-producing regimens modulated cognitive impairment, glucose metabolism, and inflammation while altering the gut microbiome. The hypothesis and the mechanism were explored in amyloid-β infused rats. Rats that received an amyloid-β(25–35) infusion into the hippocampus had either ketogenic diet (AD-KD), intermittent fasting (AD-IMF), 30 energy percent fat diet (AD-CON), or high carbohydrate (starch) diet (AD-CHO) for 8 weeks. AD-IMF and AD-CHO, but not AD-KD, lowered the hippocampal amyloid-β deposition compared to the AD-CON despite serum ketone concentrations being elevated in both AD-KD and AD-IMF. AD-IMF and AD-CHO, but not AD-KD, improved memory function in passive avoidance, Y maze, and water maze tests compared to the AD-CON. Hippocampal insulin signaling (pAkt→pGSK-3β) was potentiated and pTau was attenuated in AD-IMF and AD-CHO much more than AD-CON. AD-IMF and AD-CON had similar glucose tolerance results during OGTT, but AD-KD and AD-IMF exhibited glucose intolerance. AD-KD exacerbated gut dysbiosis by increasing *Proteobacteria*, and AD-CHO improved it by elevating *Bacteriodetes*. In conclusion, ketone production itself might not improve memory function, insulin resistance, neuroinflammation or the gut microbiome when induced by ketone-producing remedies. Intermittent fasting and a high carbohydrate diet containing high starch may be beneficial for people with dementia.

## Introduction

Alzheimer’s disease (AD) is a chronic neurodegenerative disease that results in 50–70% of dementia. The number of AD patients is increasing with increasing life expectancy since the incidence of AD increases rapidly in people over 70 years of age.^(1)^ Moreover, changing lifestyles and dietary patterns may be contributing to obesity, insulin resistance, and chronic inflammation that are believed to be risk factors for AD, although their relationship to the etiology of AD remains poorly understood.^(2)^ AD progression is characterized by the accumulation of β-amyloid plaques and neurofibrillary tangles in the brain.^(2)^ Brain insulin resistance is also involved in AD progression, and it is accompanied by increased neuroinflammation and oxidative stress in the brain.^(3,4)^ The increase of oxidative stress in AD is involved in the mitochondrial dysfunction that increases reactive oxygen species.^(5)^ Thus, oxidative stress, inflammation and insulin resistance in the brain can lead to the development of AD. These changes in the brain are reported to be connected to systemic insulin resistance.^(6)^

Obesity increases amyloid-β deposition and neurofibrillary tangles, which promote the progression of AD.^(7)^ The reduction of abdominal fat mass can be an intervention to prevent metabolic diseases, including Alzheimer’s disease in the elderly, especially in post-menopausal women. Caloric restriction is known to be beneficial for the reduction of body fat, oxidative stress, inflammation, and cognitive dysfunction.^(8,9)^ However, it is difficult to reduce body fat by decreasing calorie intake, since people have difficulty with compliance for long periods. Various measures have been used by people for losing body fat. Ketogenic diets (KD) and intermittent fasting (IMF) are recommended to promote fat utilization as an energy source. People are restricted to consuming 50–100 g of carbohydrate per day with adequate protein intake in the KD, whereas people can eat food only during certain periods in IMF.^(10,11)^ Both measures promote fat utilization in the body by making ketone bodies, which are products of incomplete oxidization of fatty acids. KD induces ketosis, but its effects on energy, glucose, and lipid metabolism are inconsistent.^(12)^ In our previous studies,^(13,14)^ IMF decreased epididymal fat pads by increasing energy expenditure in young rats but IMF, especially with HF diets, increased insulin resistance, possibly by attenuating hepatic insulin signaling.^(13)^ However, in AD-induced estrogen-deficient rats, IMF protected against the deterioration of memory function and lipid metabolism in AD-induced estrogen-deficient rats.^(14)^ However, IMF might be associated with disturbances in the gut microbiome.

The gut is home to trillions of bacterial cells and thousands of bacterial species.^(15)^ Although the microbiota in adults is fairly resilient, it can be changed within individuals by both internal (disease status) and external (diets) stimuli. The availability of nutrients for gut microbiota modify composition the gut microbiome. Persons consuming high levels of microbiota-accessible carbohydrate (MAC) display greater microbial richness and diversity as compared to those consuming low levels of MAC.^(16)^ MAC restriction reduces short-chain fatty acid (SCFA) production, the primary end products of bacterial fermentation. The composition of SCFA plays an important role in altering body metabolism. High production of acetate is reported to increase insulin resistance through the gut-brain axis and high production of propionate and butyrate act as histone deacetylase inhibitors to epigenetically influence host gene expression.^(17)^ Thus, changes in the gut microbiome are linked to not only inflammation in the gut, but also energy, glucose, and lipid metabolisms.

KD change substrate utilization in intermediary metabolism and increases the production of ketones for use as an energy source instead of carbohydrates. They can also change the gut microbiome.^(18)^ Children with severe epilepsy are sometimes treated with KD, and fiber-consuming bacteria becomes less abundant.^(19)^ Therefore, we hypothesized that interventions that increase blood ketone levels, including KD and IMF, may modulate memory function, and energy and glucose metabolisms in rats with memory impairment, and that they may also modify the gut microbiome. We examined the hypothesis in rats infused with amyloid-β into the hippocampus.

## Materials and Methods

### Animal care and surgical procedures

Male Sprague Dawley rats that weighed an average of 194 ± 16 g were purchased from Daehan Bio, Inc. (Eum-Sung, Korea) and were acclimated in the animal facility. Rats were raised in individual stainless-steel cages in a controlled environment (23°C, 12-h light/dark cycle) and they had free access to food and water. All study procedures were adopted based on the Guide for the Care and Use of Laboratory Animals from the National Institutes of Health and were approved by the Institutional Animal Care and Use Committee of Hoseo University (2014-06). After 1-week of acclimation in the animal facility, the rats were anesthetized with an intraperitoneal injection of a mixture of ketamine and xylazine (100 and 10 mg/kg, respectively which was used for all procedures requiring anesthesia) and placed in a stereotaxic device. A cannula was inserted into the bilateral CA1 subregions of the hippocampus as previously described.^(20,21)^ The cannula was connected to 22-gauge tubing filled with amyloid-β(25–35) for the four AD groups or amyloid-β(35–25) for the Non-AD-CON group; amyloid-β(35–25) was used for the Non-AD-CON group as a control because it has the reverse sequence of amyloid-β(25–35) and is not toxic and does not aggregate in the brain.^(22)^ Both types of amyloid-β were dissolved in sterile saline and infused into the bilateral CA1 subregions using an osmotic pump (Alzet Osmotic Pump Company, Cupertino, CA) at a rate of 3.6 nmol/day for 14 days. At the initial day of amyloid-β infusion, the assigned diet was provided for each group for 8 weeks.

### Diet preparation and experimental design

All rats had free access to a modified polyphenol-free AIN-93 semi-purified high-fat diet, which has been shown to induce obesity and insulin resistance.^(13)^ The normal diet was a modified semi-purified AIN-93 formulation consisting of 53 energy percent (En%) carbohydrates,^(23)^ 19 En% protein, and 28 En% fats. The ketogenic diet was composed of 0.1 En% carbohydrates, 19 En% protein, and 81 En% fats, whereas the high CHO diet contained 68 En% carbohydrates, 19 En% protein, and 13 En% fats (Table 1). The major carbohydrate, protein, and fat sources were starch plus sugar (2.4:1,w/w), casein (milk protein), and soybean oil plus lard (CJ Co., Seoul). The proper amounts of starch, casein, lard, and vitamin and mineral mixture were mixed, sifted, and stored at 4°C. The respective diet was provided by pressing the weighed powder tightly into the food container every other day, and the remaining feed was weighed and discarded.

The rats were divided into five treatment groups of 10 rats each: AD model rats that received either a 28% fat diet (normal diet) with *ad libitum* feeding (AD-CON), ketogenic diet (AD-KD), high CHO diet (AD-CHO), and normal diet with intermittent fasting (AD-IMF). The sham-operated rats received the same diet (normal diet) as AD-CON (Non-AD-CON).

### Energy expenditure

Energy expenditure was measured by indirect calorimetry during the dark phase following 6 h of fasting at 3 days after the intraperitoneal insulin tolerance test. The rats were placed in a computer-controlled metabolic chamber with a computer-monitored O_2_ and CO_2_ system and 800 ml/min airflow (Biopac Systems Inc., Goleta, CA). The CO_2_ production (VCO_2_) and O_2_ consumption (VO_2_) of the rats were measured every minute for 30 min, and the average VO_2_ and average VCO_2_ were integrated. The respiratory quotient (RQ) was calculated as VCO_2_/VO_2_ and VO_2_ and VCO_2_ values were adjusted for metabolic body size (kg^0.75^).^(13)^ Resting energy expenditure and oxidations of fat and carbohydrate were calculated as previously described.^(13)^

### Memory function by passive avoidance, Y maze, and water maze tests

At the 7th week, the rats were tested for memory deficits using a passive avoidance apparatus consisting of a two-compartment dark/light shuttle box.^(13,24)^ In the acquisition trial, electroshocks (75 V, 0.2 mA, 50 Hz) were delivered for 5 s, immediately after the rats had entered the dark chamber. Five seconds later, the rat was removed from the dark chamber and returned to its home cage. After 24 and 48 h, the retention latency time to enter the dark chamber was measured in the same way as in the acquisition trial, but electric foot shock was not delivered and the latency time was recorded to a maximum of 600 s. Shorter latencies indicate memory deficit, compared to significantly longer latencies to enter the dark room.

The next day after the passive avoidance test, rats were subjected to the Y maze test which consisted of a horizontal maze with 3 arms at 120° angles. Each arm was 50.5 cm in length, 20 cm in width, and 20 cm in height. Initially, rats were placed in one arm and then the sequences of entering into the other arms were monitored for 8 min. A right alternation was defined as a rat consecutively entering into all three arms. When each arm in the Y maze was assigned as A, B, and C, the right consecutive alternation was ABC, BCA, or CAB, but not CAC, BAB, or ABA. The spontaneous alternation (%) was calculated from the following equation: % alternation = [(Number of right alternations)/(Total number of arm entries – 2)] × 100.

Spatial memory function was assessed with a Morris water maze test, as previously described,^(22,24)^ during day 49. The Morris water maze tests hippocampal-dependent learning, including the acquisition of spatial memory. The platform was located in the zone 5 of the water pool and rats started to swim from the zone 1. The longer period to find the platform indicated memory deficiency. The point for entry of the rat into the pool and the location of the platform for the escape remained unchanged for trials on days 1, 2, and 3. On day 5, the platform was removed and the time to go to zone 5 and staying in zone 5 were measured. The test was conducted with a cut-off time of 600 s.

### Glucose metabolism and sample collection

At week 8 of the experiment, an oral glucose tolerance test was conducted after an overnight fast using an oral administration of glucose (2 g/kg). During the oral glucose tolerance test, serum glucose concentrations were measured every 10 min until 90 min and again at 120 min, and serum insulin levels were measured at 0, 20, 40, 60, 90, and 120 min. The serum glucose and insulin concentrations were measured using a Glucometer (Accuchek; Roche Diagnostics, Basel, Switzerland) and enzyme-linked immunosorbent assay (ELISA; Crystal Chem, Elk Grove Village, IL), respectively. At 3 days after the oral glucose tolerance test, an intraperitoneal insulin tolerance test was conducted after the food was removed for 6 h. The serum glucose levels were measured every 15 min for 90 min after an intraperitoneal injection of insulin (0.75 U/kg body weight).

After two days from the intraperitoneal insulin tolerance test, blood was collected from anesthetized rats by cardiac puncture, and the serum was separated by centrifugation at 3,000 rpm for 20 min. After blood collection, human insulin (5 U/kg body weight) was injected through the inferior vena cava to determine insulin signaling. Serum and tissue samples were stored at –70°C for biochemical analysis. The epididymal and retroperitoneal fat masses and uteri were then removed and weighed. HOMA-IR was calculated as follows: serum insulin (µU) × serum glucose (mmol/L)/22.5. Serum tumor necrosis factor-α (TNF-α) concentrations were measured using ELISA kits from eBioscience (San Diego, CA).

### Realtime quantitative reverse transcriptase polymerase chain reaction (RT-PCR)

Hippocampal tissues were randomly excised from 4 rats from each group and total RNA was isolated using a monophasic solution of phenol and guanidine isothiocyanate (Trizol reagent; Invitrogen, Rockville, MD). The cDNA was synthesized from total RNA with superscript III reverse transcriptase and high fidelity Taq DNA polymerase (1:1:1, v:v:v) by reverse transcription reaction in PCR. The synthesized cDNA was mixed with the primers of the genes of interest and sybergreen mix and their expressions were analyzed using a realtime PCR machine (BioRad Laboratories, Hercules, CA). The primers of TNF-α, interleukin (IL)-1β, and β-actin were given in previous studies.^(13,22)^ The expression levels genes of interest were quantified using the cycle of threshold method.^(25)^

### Immunoblot analysis

Hippocampal tissues from four rats from each group were dissected as previously described.^(22)^ Each tissue was lysed with RIPA lysis buffer with added protease inhibitors, and their protein contents were measured using a Bio-Rad protein assay kit (Hercules, CA). The lysates with equivalent amounts of protein (30–50 µg) were resolved into sodium dodecyl sulfate-polyacrylamide gel electrophoresis and the amounts of proteins of interest were examined by immunoblotting with the specific antibodies as follows: protein kinase B (PKB or Akt), phosphorylated PKB^Ser473^, forkhead box protein O1 (FOXO1), phosphorylated FOXO1^Ser256^, phosphorylated tau^Ser396^, tau (Cell Signaling Technology, Danvers, MA) and β-actin (Santa Cruz Biotech, Dallas, TX). The intensity of the proteins detected were measured using Imagequant TL (Amersham Biosciences, Piscataway, NJ).

### Next-generation sequencing gut microbiome and bacterial sequence processing

The gut microbiome community was examined from the feces by analyzing metagenome sequencing using next-generation sequencing procedures.^(26)^ Bacterial DNA was extracted from the feces of each rat using a Power Water DNA Isolation Kit (Qiagen, Valencia, CA) according to the manufacturer’s instructions. The DNA was amplified with 16S amplicon primers (V3 and V4 region) using PCR and each library was prepared using the PCR products according to the GS FLX plus library prep guide.^(26)^ The emulsion of DNA capture beads and library-beads was dispensed into a 96-well plate and the PCR amplification program was run with 16S universal primers in the FastStart High Fidelity PCR System (Roche, Basel, Switzerland) according to the manufacturer’s recommendations. Sequencing of bacterial DNA in the feces was measured by using the Illumina MiSeq standard operating procedure by a Genome Sequencer FLX plus (454 Life Sciences) in Macrogen Ltd. (Seoul, Korea).

The 16S amplicon sequences were processed using Mothur ver. 1.36. We followed the Miseq SOP to identify the taxonomy and counts of the bacteria in each fecal sample. We aligned the sequences using Silva reference alignment ver. 12350. In a preclustering step, the sequences with an identity ≥99% were merged. The chimeric sequences were detected and discarded by UCHIME. All the sequences were assigned with taxonomic classifications using Greengenes 13_8_99 and the sequences classified as mitochondria, eukaryota or unknown were removed. We conducted the picking of operational taxonomic units (OTUs) delimited at 98% identity, which was taxonomically classified by consensus using Greengenes 13_8_99. A relaxed neighbor-joining tree with one representative sequence per OTU was obtained with Clearcut after calculating uncorrected pairwise distances between aligned reads.

### Statistical analysis

All statistical analyses were conducted using SAS ver. 7 (SAS Institute, Cary, NC). All results are expressed as means ± SD. One-way analysis of variance was used to compare the groups when the results were measured only once at the end of the experiment. Multiple comparisons among the groups were conducted using Tukey’s test. Principle coordinate analysis (PCoA) in gut bacteria was analyzed by analysis of molecular variance in Mothur. A *p* value <0.05 was considered as statistically significant.

## Results

### Cognitive function

**Fig. 1:**
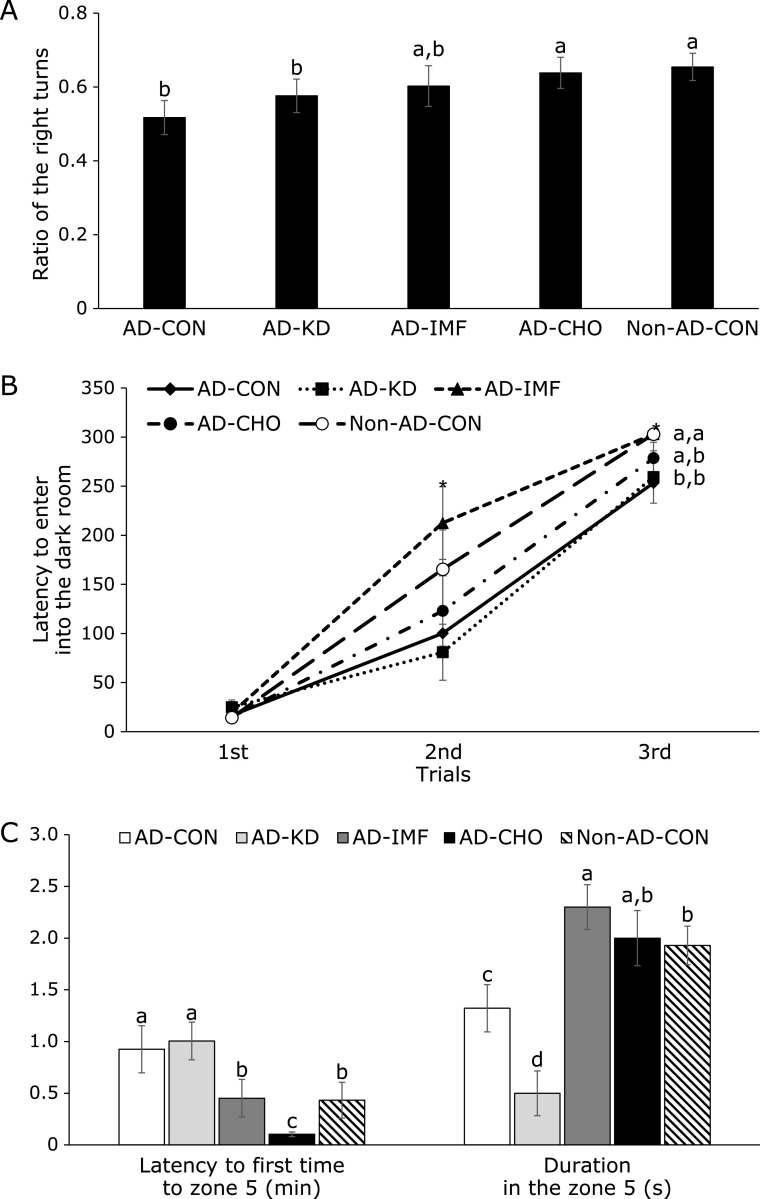
Memory deficit of rats with amyloid-β infusion. Ratio of the right turns in Y maze (A), latency time to enter the dark room in passive avoidance test at 3 trials (B), the latency time to locate zone with the platform and the period to stay in the platform zone on day 5 during the water maze test (C) were shown. The amyloid-β(25–35) infused diabetic rats AD model rats received assigned diet for 8 weeks as follows: 1) a normal diet (28 energy % fat diet) with *ad libitum* feeding (AD-CON), 2) ketogenic diet (AD-KD; 0.1% carbohydrate diet), 3) high carbohydrate diet (AD-CHO; 68 energy % carbohydrate diet) and 4) normal diet with intermittent fasting (AD-IMF). The sham-operated rats received the same diet (normal diet) as AD-CON (Non-AD-CON) for 8 weeks. Each dot and bar represents means ± SD (*n* = 10). *Significantly different among the groups by one-way-ANOVA at *p*<0.05. ^a,b,c,d^Different letters on the bars indicate significant differences by Tukey test at *p*<0.05.

Rats usually make right turns in the Y maze and then go to the next arm instead of going back to the previous arm. Rats in the AD-CON had a lower ratio of right turns to the total movements than the Non-AD-CON. Rats in the AD-IMF and AD-CHO had a higher ratio than AD-CON, and the ratio was similar to the Non-AD-CON (Fig. 1A).

In the 1st passive avoidance trial all rats entered into the darkroom and they had a small electric shock. Rats in the AD-CON had a shorter latency compared to those in the Non-AD-CON, indicating that AD-CON entered into the darkroom after forgetting the electric shock. AD-IMF delayed the latency to enter the darkroom in the 2nd trial compared to the other AD groups, and it was similar to Non-AD-CON (Fig. 1B). In 3rd trial rats in the AD-IMF and Non-AD-CON did not enter the darkroom. AD-CHO increased the latency to enter the dark room but it was quicker than that of Non-AD-CON. AD-KD did not delay the latency compared to the AD-CON (Fig. 1B). Thus, AD-IMF was beneficial for enhancing short-memory and AD-CON had partial improvement.

In the 4th trial of the water maze test to determine the spatial memory, AD-CON rats showed a delayed latency time to locate the zone 5 where the platform was located in comparison to the Non-AD-CON (Fig. 1C). AD-KD did not improve the latency period to go to zone 5, and AD-IMF shortened the latency time to go to zone 5 as much as the Non-AD-CON. Interestingly, AD-CHO went to zone 5 more quickly than the Non-AD-CON (Fig. 1C). During the 4th water maze trial, the platform was removed, and rats were hunting for the platform. If rats remembered the platform area, they would stay longer in zone 5. The duration in zone 5 was measured. Rats in the AD-CON stayed in zone 5 for less time than those in the Non-AD-CON and rats, but the time in zone 5 was shortest for the AD-KD among all the groups. Rats in the AD-IMF group stayed longer in the zone 5 than those of Non-AD-CON (Fig. 1C). Rats in the AD-CHO stayed in zone 5 for a similar time as in the Non-AD-CHO. These results about memory function showed that AD-IMF improved short- and spatial-memory function the most and AD-CHO had some improvement.

### Amyloid-β deposition and brain insulin signaling

**Fig. 2:**
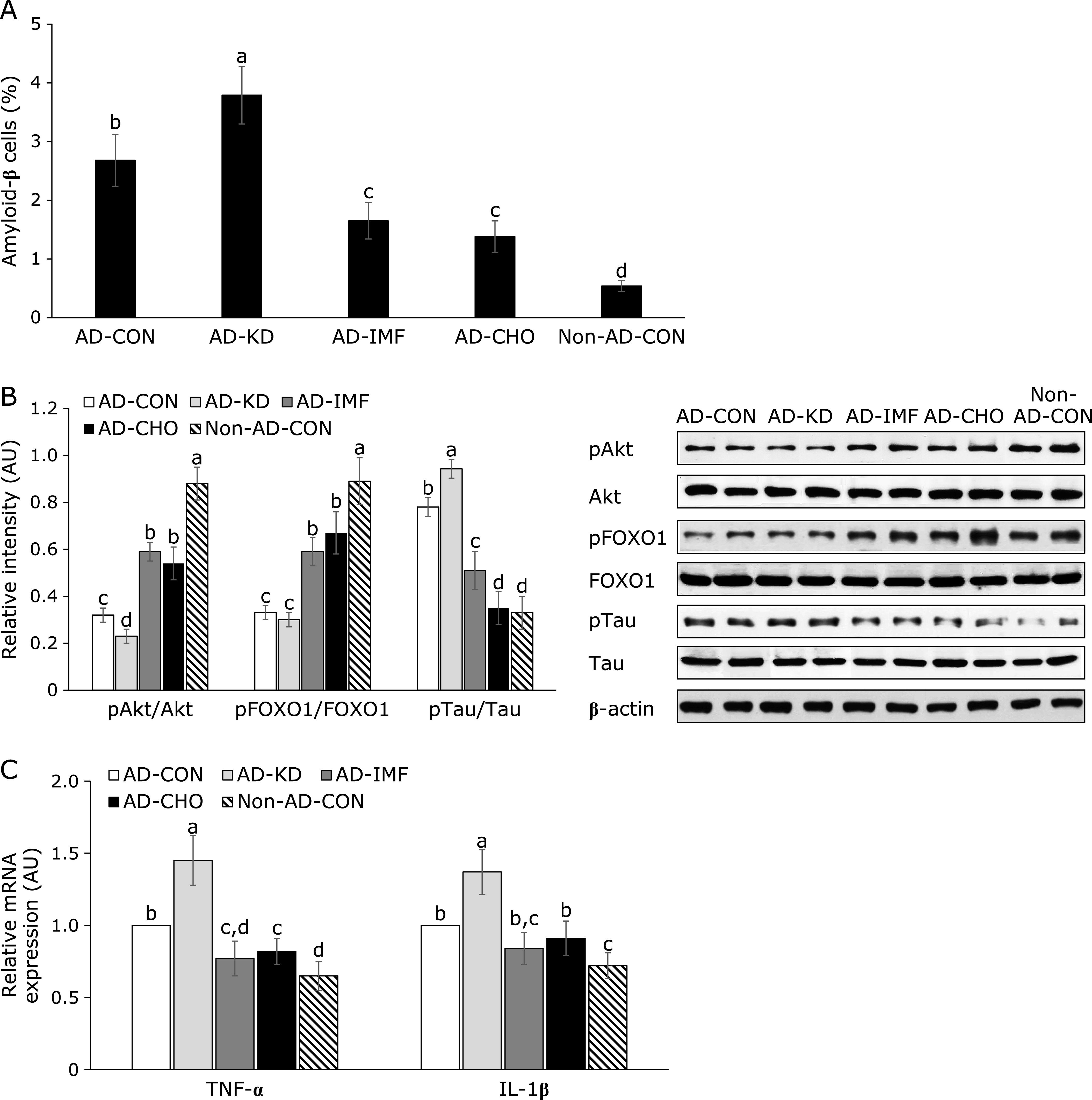
Insulin signaling and cytokines in the hippocampus. After preparing lysates of the hippocampus, amyloid-β was stained by anti-amyloid-β antibody in brain section and the percentage of amyloid-β stained cells (A) was given (*n* = 5). The phosphorylation and expressions of proteins related to insulin signaling (B) was measured with western blot analysis and relative intensity was calculated based on designated protein contents (*n* = 4). The amyloid-β(25–35) infused diabetic rats AD model rats received assigned diet for 8 weeks as follows: 1) a normal diet (28 energy % fat diet) with *ad libitum* feeding (AD-CON), 2) ketogenic diet (AD-KD; 0.1% carbohydrate diet), 3) high carbohydrate diet (AD-CHO; 68 energy % carbohydrate diet) and 4) normal diet with intermittent fasting (AD-IMF). The sham-operated rats received the same diet (normal diet) as AD-CON (Non-AD-CON) for 8 weeks. Each dot and bar represents means ± SD. ^a,b,c,d^Different letters on the bars indicate significant differences by Tukey test at *p*<0.05.

 The amyloid-β accumulation was elevated in AD-CON compared to Non-AD-CON and it further increased in AD-KD compared to the AD-CON. AD-IMF and AD-CHO lowered AB accumulation (Fig. 2A). The phosphorylation of Akt was much lower in the AD-CON than Non-AD-CON and AD-KD decreased the phosphorylation compared to the AD-CON. AD-IMF and AD-CHO increased the Akt phosphorylation compared to the AD-Con, but they did not increase it as much as the Non-AD-CON (Fig. 2B). The phosphorylation of FOXO-1, a downstream signal of Akt, was much lower in AD-CON than Non-AD-CON and AD-KD was similar to AD-CON. AD-IMF and AD-CHO elevated the phosphorylation of FOXO1 compared to the AD-CON, but they did not increase it similar to the Non-AD-CON (Fig. 2B). The phosphorylation of tau, an indicator of amyloid-β deposition, increased in AD-CON compared to the Non-AD-CON. AD-KD exacerbated tau phosphorylation. Tau phosphorylation was lower in AD-IMF and AD-CHO than AD-CON (Fig. 2B).

Hippocampal TNF-α and IL-1β mRNA expression was higher in the AD-CON than the Non-AD-CON (Fig. 2C). Their expressions were lower in AD-IMF and AD-CHO, but they increased in AD-IMF, compared to the AD-CON (Fig. 2C).

### Energy metabolism

Body weight gain was not significantly different between AD-CON and Non-AD-CON, and AD-KD did not modulate body weight gain from the AD-KD. However, AD-CHO and AD-IMF significantly lowered body weight gain compared to AD-CON, and AD-IMF decreased it more than AD-CHO (Table 2). Visceral fat mass was determined by summing the weight of retroperitoneal fat and epididymal fat mass. Visceral fat mass was not significantly different between AD-CON and Non-AD-CON and its mass in the AD-KD did not differ from the AD-CON (Table 2). However, AD-IMF and AD-CHO decreased the visceral fat mass compared to the AD-CON. In visceral fat mass retroperitoneal fat and epididymal fat mass showed the similar patterns.

Weight gain and visceral fat mass are altered by energy intake and energy expenditure (Table 2). Energy intake was similar in both of AD-CON and Non-AD-CON. Energy intake in the AD-KD, CHO and AD-IMF groups lowered energy intake compared to the AD-CON and that in the AD-IMF was lowest among the groups. Energy expenditure tended to be lower in Non-AD-CON than AD-CON (*p* = 0.06) whereas energy expenditure in the other experimental groups of the AD rats did not differ significantly from that in the AD-CON. Energy expenditure was significantly higher in Non-AD-CON than the AD-IMF, AD-CHO, and AD-KD groups (Table 2). RQ did not significantly differ between AD-CON and Non-AD-CON, and it was significantly lower in the AD-KD than AD-CHO. According to RQ, daily energy expenditure and body weight, carbohydrate, and fat oxidation as fuel sources were significantly different among the groups. Carbohydrate oxidation was significantly higher in AD-CON than Non-AD-CON and it was lowered in the descending order of AD-CHO, AD-CON, and AD-IMF = AD-KD (Table 2). In contrast to carbohydrate oxidation, fat oxidation was higher in the Non-AD-CON than AD-CON and among AD rats, it was higher in the ascending order of AD-CHO, AD-CON, and AD-IMF = AD-KD (Table 2). These results suggested that AD increased the utilization of carbohydrate more than fat as a fuel and that IMF and AD-KD suppressed the utilization of carbohydrate.

### Glucose metabolism

Serum β-hydroxy butyrate concentrations were not significantly different between AD-CON, AD-CHO, and Non-AD-CON, but the concentrations were higher in AD-KD and AD-IMF than AD-CHO (Table 3). Serum β-hydroxy butyrate concentrations in AD-KD was much higher than AD-IMF (Table 3). Overnight-fasting serum glucose concentrations were not significantly different among the groups (Table 3). However, serum insulin levels at fasting state were higher in the AD-CON than Non-AD-CON, and they were higher in AD-IMF in comparison to AD-CON and AD-CHO (Table 3). As calculated by serum glucose and insulin levels at fasting state, HOMA-IR was higher in the ascending order of AD-CHO, Non-AD-CON, AD-CON = AD-KD, and AD-IMF and it were similar between AD-CHO and Non-AD-CON (Table 3).

**Fig. 3:**
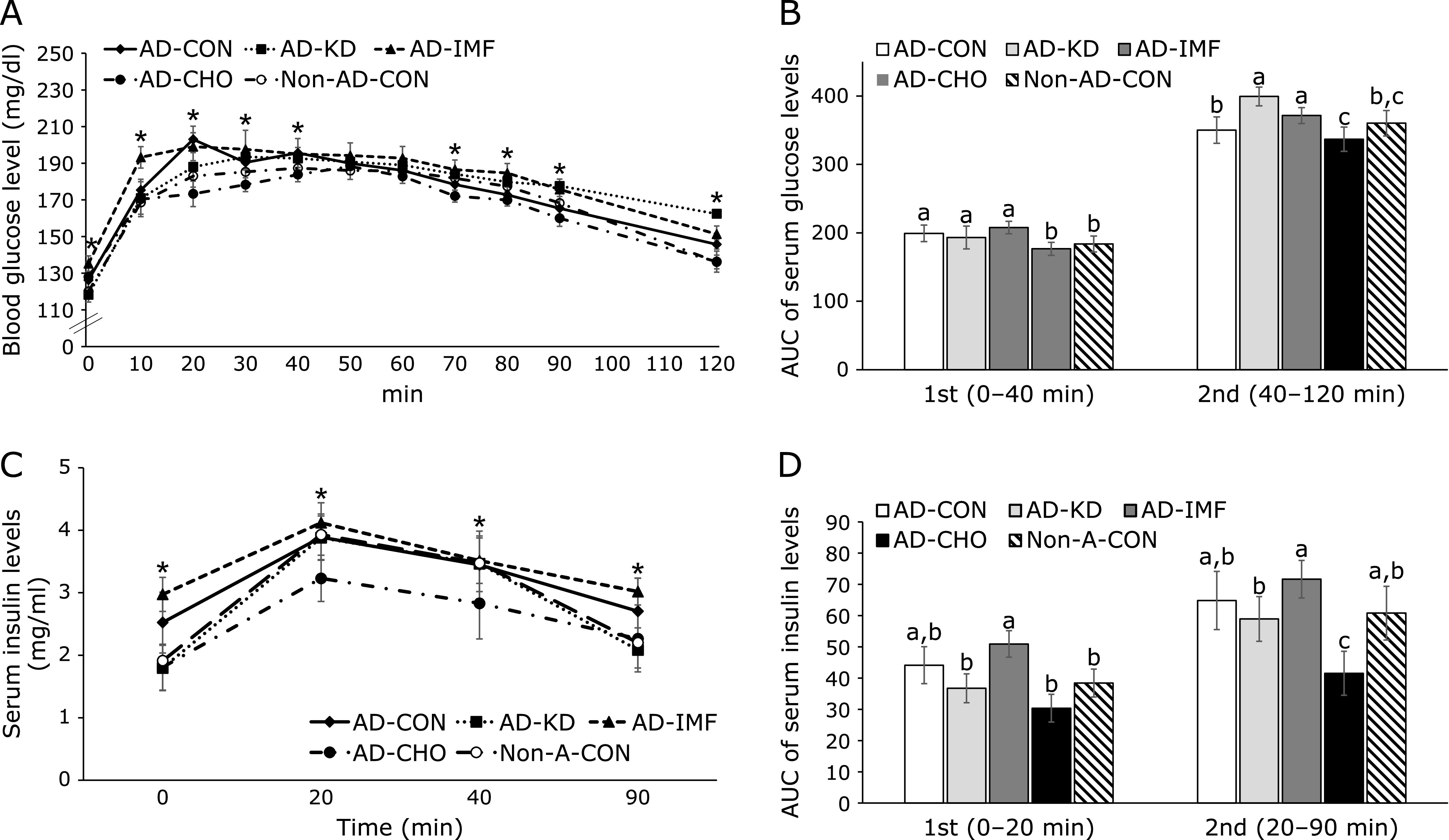
Oral glucose tolerance test. Glucose (2 g/kg body weight) was orally administered to the rats. Serum glucose levels (A) and serum insulin levels (C) were also measured at the assigned times after glucose challenge. Area under the curve (AUC) of glucose (B) and insulin (D) was calculated in the first (0–40 min) and second phases (40–120 min). The amyloid-β(25–35) infused diabetic rats AD model rats received assigned diet for 8 weeks as follows: 1) a normal diet (28 energy % fat diet) with *ad libitum* feeding (AD-CON), 2) ketogenic diet (AD-KD; 0.1% carbohydrate diet), 3) high carbohydrate diet (AD-CHO; 68 energy % carbohydrate diet) and 4) normal diet with intermittent fasting (AD-IMF). The sham-operated rats received the same diet (normal diet) as AD-CON (Non-AD-CON) for 8 weeks. Each dot and bar represents means ± SD (*n* = 10). *Significantly different among the groups by one-way-ANOVA at *p*<0.05. ^a,b,c^Different letters on the bars indicate significant differences by Tukey test at *p*<0.05.

Serum glucose levels were increased until the peak level in the AD-CON compared to the Non-AD-CON. Among different interventions, the peak levels were lower in AD-CHO than AD-IMF and AD-KD (Fig. 3A). The 1st phase AUC of serum glucose levels during OGTT was much higher in the AD-CON than the Non-AD-CON and it slightly increased in the AD-IMF compared to AD-CON. The AUC at the 1st phase was similar between AD-CHO and Non-AD-CON. After 40 min during OGTT serum glucose levels were slowly decreased in all rats, but the slope of the decrease in the AUC was lower in AD-CHO than AD-KD and AD-CON (Fig. 3B).

After reaching the peak, serum glucose concentrations decreased in all rats, but the slope of decrease was different among the groups. Serum glucose levels decreased more slowly in the AD-CON than the Non-AD-CON. Among the interventions, AD-KD lowered serum glucose levels most slowly, and AD-IMF also decreased the levels more slowly than AD-CON (Fig. 3A). The area under the curve (AUC) of serum glucose levels in the 1st part was higher in the AD-CON than the Non-AD-CON, and it was lowered in descending order of AD-IMF, AD-CON, AD-KD, and AD-CHO = Non-AD-CON. The 2nd parts of OGTT was higher in the AD-CON than the Non-AD-CON, and it increased in AD-IMF and AD-KD and decreased AD-CHO compared to the AD-CON (Fig. 3B). Serum insulin levels were not significantly different between AD-CON and Non-AD-CON and the levels at 20 and 40 min were similar among AD-CON, AD-KD, and AD-IMF (Fig. 3C and D). They were much lower in AD-KD than AD-CON (Fig. 3C and D).

**Fig. 4:**
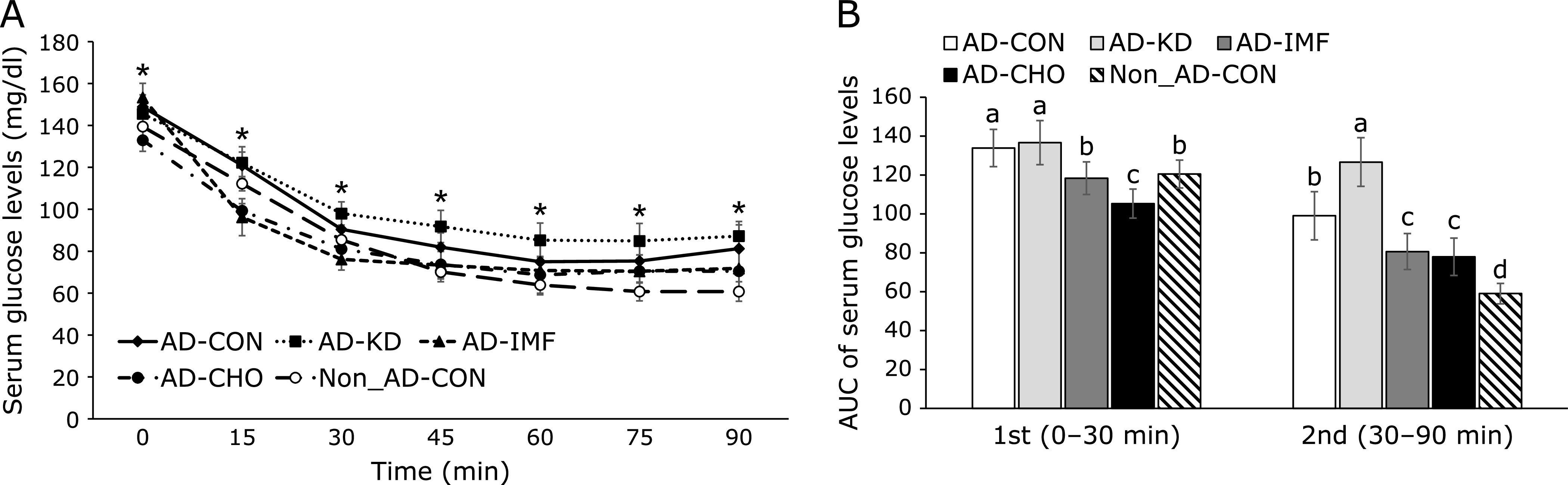
Changes in the serum glucose levels during an intraperitoneal insulin tolerance test (IPITT). An IPITT was conducted by intraperitoneally injecting insulin (1 U/kg body weight) after a 6 h fast. The serum glucose levels (A) and area under the curve of serum glucose levels (B) were measured. The amyloid-β(25–35) infused diabetic rats AD model rats received assigned diet for 8 weeks as follows: 1) a normal diet (28 energy % fat diet) with *ad libitum* feeding (AD-CON), 2) ketogenic diet (AD-KD; 0.1% carbohydrate diet), 3) high carbohydrate diet (AD-CHO; 68 energy % carbohydrate diet) and 4) normal diet with intermittent fasting (AD-IMF). The sham-operated rats received the same diet (normal diet) as AD-CON (Non-AD-CON) for 8 weeks. Each dot and bar represents means ± SD (*n* = 10). *Significantly different among the groups by one-way-ANOVA at *p*<0.05. ^a,b,c,d^Different letters on the bars indicate significant differences by Tukey test at *p*<0.05.

After intraperitoneal injection of insulin, serum glucose concentration in rats in the AD-CON decreased until 30 min and lower serum glucose levels were maintained from 60–90 min compared to the Non-AD-CON, and no rebounce was detected in the Non-AD-CON (Fig. 4A). Rats in the AD-KD exhibited the highest serum glucose levels during IPITT among the groups (Fig. 4A). AD-KD decreased the response to insulin and it may induce hyperglycemia when glucose availability increases. However, serum glucose levels in the 1st part of IPITT of the AD-IMF quickly decreased until 30 min, and then the levels were maintained in comparison to the rats with AD-CON groups (Fig. 4A). Rats in the CHO group exhibited rapidly decreased serum glucose levels similar to the AD-IMF (Fig. 4A). AUC at 1st and 2nd parts was greater in the AD-CON than Non-AD-CON (Fig. 4B). AUC of the 1st part of IPITT was higher in the ascending order of AD-CHO, Non-AD-CON = AD-IMF, and AD-CON = AD-KD. However, AUC of the 2nd part was much greater in AD-KD; thus rats with AD-IMF and AD-CHO showed lower serum glucose levels than those with AD-CON (Fig. 4B). The results suggested that AD-CON were insulin resistant and that AD-IMF and AD-CHO decreased the insulin resistance at high serum glucose levels when insulin was high.

### Lipid metabolism

Serum total and HDL cholesterol levels did not differ between the AD-CHO and Non-AD-CON but serum LDL and triglyceride concentrations were higher in the AD-CON than the Non-AD-CON (Table 3). Interestingly, AD-KD lowered serum total and LDL concentrations and triglyceride concentrations and increased HDL concentrations in comparison to the AD-CON (Table 3). However, AD-CHO increased total and LDL cholesterol and triglyceride concentrations compared to the AD-CON, and it decreased serum HDL cholesterol concentrations (Table 3). AD-IMF did not alter lipid profiles in the circulation compared to the control. The results suggested that AD-CON rats exhibited some dyslipidemia and that AD-CHO worsened cholesterol metabolism and AD-KD rather improved it (Table 3).

### Gut microbiome

**Fig. 5:**
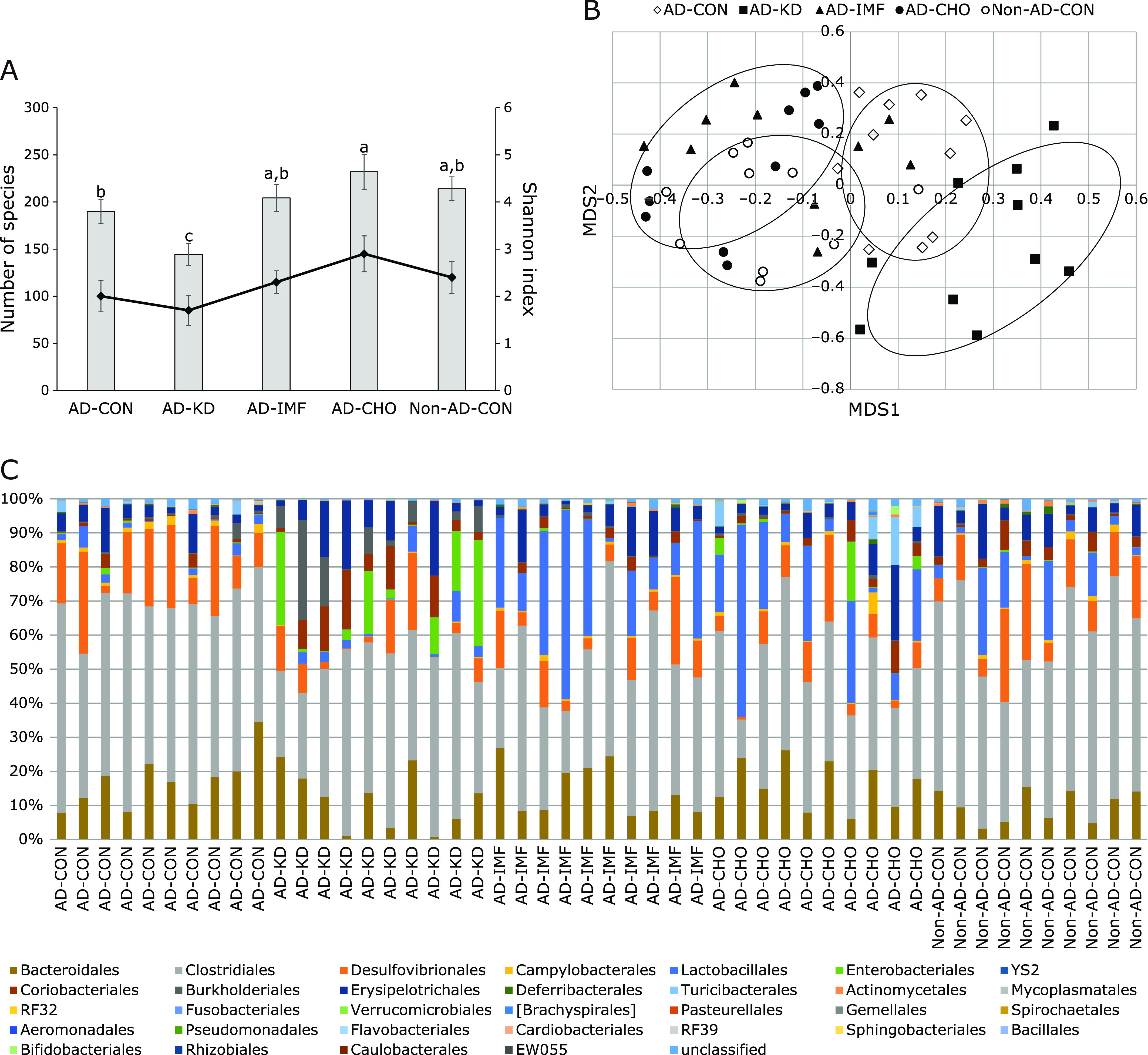
The profiles of gut microbiomes. Feces were collected from the cecum at the end of experimental periods and the bacterial DNA was analyzed. The number of species and Shannon index (A), the fecal bacterial community by principal coordinate analysis (PCoA) (B) and proportion of taxonomic assignments [order] for gut microbiomes (C) was determined from the feces. The amyloid-β(25–35) infused diabetic rats AD model rats received assigned diet for 8 weeks as follows: 1) a normal diet (28 energy % fat diet) with *ad libitum* feeding (AD-CON), 2) ketogenic diet (AD-KD; 0.1% carbohydrate diet), 3) high carbohydrate diet (AD-CHO; 68 energy % carbohydrate diet) and 4) normal diet with intermittent fasting (AD-IMF). The sham-operated rats received the same diet (normal diet) as AD-CON (Non-AD-CON) for 8 weeks. Each dot and bar represents means ± SD (*n* = 10). ^a,b,c^Different letters on the bars indicate significant differences by Tukey test at *p*<0.05.

The number of bacterial species did not differ between AD-CON and Non-AD-CON whereas AD-CHO tended to increase the number of species (*p* = 0.063) and AD-KD significantly decreased it. The Shannon index, an index of the richness of gut microbiome, showed the same tendency as the number of species. Shannon index was much lower in AD-KD than AD-CON (Fig. 5A). Principal coordinate analysis (PCoA) shows the clustering of the gut bacterial community in the groups, and there was a significant difference among the groups (*p* = 0.012; Fig. 5B). The AD-CON group exhibited a separation of the community from the Non-AD-CON with exception of one group. AD-CHO showed a separating cluster form AD-CON which was shared with Non-AD-CON. AD-IMF had sharing with AD-CON and Non-AD-CON. AD-KD had a separated cluster with Non-AD-CON and AD-CON (Fig. 5B). Thus, AD-CON somewhat changed the gut bacterial community from Non-AD-CON and AD-KD and AD-CHO influenced the gut microbiome in AD rats.

The significant differences of the gut microbiota community among all groups were examined by analysis of molecular variance (*p* = 0.022). At the Order level, the relative counts of *Clostridales* were higher in AD-Con than Non-AD-CON, but those of *Bacteroidales* were not significantly different between the two groups (Fig. 5C). AD-IMF decreased *Clostridales* and had a tendency to increase *Lactobacillales*. However, AD-KD increased the relative counts of *Proteobacteria* especially *Enterobacteriales* compared to other groups (Fig. 5C).

## Discussion

Ketones are produced as a consequence of low carbohydrate diets, intermittent fasting, and very low calorie diets. However, the interventions using ketogenic diet and intermittent fasting may have similar or different effects on memory function when they produce ketones. Ketone utilization can reduce the production of reactive oxygen species by modulating the ratio of oxidized and reduced form of NAD to improve mitochondrial function.^(27)^ We hypothesized that dietary interventions that increase ketone production may modulate mild cognitive impairment, glucose metabolism, inflammation, and the gut microbiome in amyloid-β infused rats, and the mechanisms involved were explored. A high CHO diet was also included since it is an opposite intervention from the ketogenic diet. Hippocampal insulin resistance and neuroinflammation were evaluated in the present study studied since they are known to impair memory function. AD-KD and AD-IMF differently influenced memory function, although both increased serum ketone levels. AD-IMF improved memory function and but AD-KD did not protect against memory impairment. AD-IMF potentiated hippocampal insulin signaling (pAkt→pGSK-3β) and reduced mRNA expression of TNF-α and IL-1β, inflammatory markers, in the hippocampus but AD-KD rather impaired them. The changes of insulin sensitivity and inflammation are the brain is known to be associated with gut microbiome. AD-KD induced gut dysbiosis compared to the Non-AD-CON and AD-IMF did not alter gut microbiome from the Non-AD-CON: AD-KD disrupted gut microbiome composition by increasing *Proteobacteria* and lowering diversity, but AD-IMF improved it. AD-CHO had an overlapped cluster of the gut microbiome with Non-AD-CON and it enhanced memory function. Therefore, the AD-KD increased amyloid-β in the hippocampus more than the AD-CON by attenuating hippocampal insulin signaling and increasing neuroinflammation. The AD-IMF and AD-CHO prevented the attenuation of insulin signaling and inflammation in the hippocampus and the prevention of gut dysbiosis might be involved in the process. The IMF and CHO diet patterns might be beneficial for protecting against the development of Alzheimer’s disease.

The ketogenic diet is a very low-carbohydrate and high-fat diet that mimics the fasting state. It is known to have a beneficial effects on epileptic seizures that develop due to abnormally excessive neuronal activity in the brain.^(28)^ The seizures are accompanied by uncontrolled shaking movements. KD reduces the symptoms of seizure by using ketone instead of glucose in the brain. The KD effects on cognitive function and brain growth have been researched, but the results are contradictory. The KD was shown to have harmful effects on memory function and brain growth,^(29)^ but another study demonstrated beneficial effects on brain function.^(30)^ Ketones, acetoacetate, and β-hydroxy butyrate are alternative energy sources to glucose. Persons with Alzheimer’s disease have an impairment of glucose uptake in the brain, but ketone utilization in the brain appears not to be impaired.^(31)^ The different results may be associated with types of fat utilized in the ketogenic diet. Medium-chain fat intake has better effects on epilepsy and neurodegenerative diseases. However, intermittent fasting has been reported to improve memory function, although both the ketogenic diet and intermittent fasting elevate serum ketone levels by producing ketones in the liver.

The main characteristics of Alzheimer’s disease are amyloid-β deposition in the hippocampus and impairment of memory function.^(32)^ The mechanism of amyloid-β accumulation in the hippocampus remains unclear. The changes of amyloid precursors into amyloid-β and tangles of amyloid-β in the hippocampus are involved in the development and progression of Alzheimer’s diseases.^(32)^ The major cause is the attenuation of hippocampal insulin signaling and potentiation of tau phosphorylation.^(33)^ The brain insulin sensitivity was partly consistent with peripheral insulin sensitivity.^(34)^ IMF, but not KD, improved systemic insulin sensitivity measured by IPITT but HOMA-IR, representing insulin resistance in the fasting state, was higher in AD-IMF than AD-KD in the present study. Furthermore, hippocampal insulin signaling and neuroinflammation are reported to be crucial to preventing and alleviating amyloid-β deposition in an animal model using infused amyloid-β into the hippocampus.^(24,35,36)^ AD-IMF improved hippocampal insulin signaling and neuroinflammation but its ketone production was not as much as AD-KD in the present study. This present study revealed that AD-IMF and AD-CHO potentiated insulin signaling and reduced neuroinflammation in the present study but not AD-KD. The lack of benefit appeared to be related to its effects on the gut microbiome. AD-KD induced gut dysbiosis, which may have increased systemic and neuronal inflammation as shown in previous studies.^(37,38)^ Therefore, ketone production may help provide energy, but it also may impair insulin signaling and neuroinflammation in the hippocampus and increase the amyloid-β deposition in the hippocampus. Improvements in insulin sensitivity and inflammation in the brain and peripheral tissues protect against memory dysfunction.

The gut microbiome is another factor that influences various neurological diseases through the bidirectional microbiota-gut-brain axis.^(19)^ The gut microbiota influence host memory function and neurodegenerative diseases. The gut microbiome is altered in patients with Alzheimer’s disease.^(39,40)^ They have less *Bacteroidetes* and *Bifidobacterium*, and more *Firmicutes* than healthy controls but some studies have shown different results.^(39,40)^ Gut microbiota dysbiosis increases the permeability of both the gut and blood-brain barrier, and they secrete large amounts of amyloids and lipopolysaccharides that contribute to the production of proinflammatory cytokines.^(41)^ Dysbiosis may mediate the pathogenesis of Alzheimer’s disease and other neurodegenerative disorders by inflammation driven pathogenesis.^(38)^ The present study demonstrated that rats that developed AD-like pathologies had gut microbiome dysbiosis as evidenced by decreased relative counts of *Lactobacillales*, but without altering *Bacteriodetes*. Therefore, patients with AD may also have dysbiosis which induces neuroinflammation which in turn exacerbates memory dysfunction.

The KD has been used for modulating epilepsy treatment.^(19)^ KD decreases the relative abundance of *Bifidobacterium longum* by 3.4 folds and *B. adolescentis *by 16 folds, *Eubacterium rectale *by 5 folds and *Dialister* by 5.5 folds and increases the relative abundance of *Escherichia coli* by 2.7 folds in children with severe epilepsy.^(19)^ KD diminished the relative abundance of health-promoting and fiber-consuming bacteria. KD effects on the gut microbiome remains unclear. A KD containing 4.8% fiber and 3.2% carbohydrates increased the relative abundance of putatively beneficial gut microbiota (*Akkermansia* *muciniphila* and *Lactobacillus*) and reduced that of putatively pro-inflammatory taxa (*Desulfovibrio* and *Turicibacter*) in young healthy mice.^(18)^ The present study demonstrated that KD increased the relative counts of *Proteobacteria*, especially *Enterobacteriales* whereas IMF decreased *Clostridales* and had a tendency to increase *Bacteroidales*. The KD was included no CHO, 3.4% cellulose and lard for fat source in the present study. Thus, the composition of KD might affect the changes of gut microbiome.

High CHO diets lead to metabolic syndrome, especially dyslipidemia in Asians who have consumed very high CHO and very low fat diets.^(42)^ However, high CHO diets improved memory function better than KD in the present study. Furthermore, AD-CHO improved memory function better than AD-KD and AD-CON. AD-CHO shared the bacterial community with Non-AD-CON and AD-IMF. AD-CHO increased *Lactobacillales* and decreased *Clostridales* similar to AD-IMF. Thus, carbohydrate in the diet may be utilized by gut microbiota, although it is known to be mostly absorbed in the small intestine. Starch in the diet may act as a resistant starch in the animal study since high CHO diet acts as an anti-obesity food compared to a high fat diet.^(43)^ However, no study has examined a high CHO diet (mainly high in starch) on the gut microbiome, although a high fat diet induced microbiome dysbiosis.^(44,45)^ Microbiome available carbohydrates including resistant starch and soluble dietary fiber improve the gut microbiome composition.^(46)^ A high carbohydrate diet may influence gut microbiome possibly by providing some carbohydrate to the gut microbiota in rats.

There are some limitations to this study, first animal models are not fully applicable to humans. Additionally, although the AD model used in this study mimics human AD in many respects, the etiology of the disease is different in the rat model than in humans. In this study, the animal model was generated by the injection of amyloid-β, not by spontaneously making amyloid-β in the brain. The deposition of amyloid-β tangles in the hippocampus was modulated by different diets. Finally, the diets used were semi-synthetic diets that are different from eating whole foods and may have different effects on the microbiome. However, despite the limitations of the study, the results suggest ways of preventing or delaying human AD that should be studied in humans.

In conclusion, ketone production itself might not improve memory function and the gut microbiome. KD impaired memory function compared the AD-CON, and it increased the ratio of *Proteobacteria*, especially *Enterobacteriales*, which are relatively harmful bacteria. KD needs to include sufficient microbiome available carbohydrates and optimal fat composition by modifying fat types and including non-digestible carbohydrates should be considered. IMF improved memory function and gut microbiomes, although it contained the same amounts of cellulose. Therefore, IMF and high CHO diets might improve memory function and energy and glucose metabolism. Intermittent fasting might be conducted by having long over-night fasting (16–17 h fasting) after consuming late lunch (about 3–4 PM). It can be applicable in clinical practices. It might be adopted by nursing homes, assisted living facilities, and programs like “Meals on Wheels” which provide meals for elderly people living at home.

## Author Contributions

SP conceived of the study, contributed to the design and prepared the initial draft of the manuscript. TZ and JYQ carried out animal study and the statistical analysis, and TZ and XW biochemical assays. All authors read, made suggestions and approved the final version. All authors discussed the final results and approved the final manuscript.

## Figures and Tables

**Table 1:** The composition of experimental diets (Unit: g/kg diet)

	Normal diet	Ketogenic diet	High carbohydrate diet
Corn starch	371	1	470
Sucrose	200	1	200
Casein	210	320	190
Lard	115	574	36
Soybean oil	20	20	20
Cellulose	34	34	34
AIN-76 mineral mixture	35	35	35
AIN-76 vitamin mixture	10	10	10
Choline chloride	2	2	2
dl-Methionine	3	3	3
Cholesterol	25	25	25

**Table 2:** Body weight, energy intake, energy expenditure and inflammation index

	AD-CON (*n* = 10)	AD-KD (*n* = 10)	AD-IMF (*n* = 10)	AD-CHO (*n* = 10)	Non-AD-CON (*n* = 10)
Final body weight (g)	366 ± 27^ab^	353 ± 32^b^	291 ± 27^d^	317 ± 26^c^	373 ± 15^a^
Body weight gain (g)	169 ± 12^a^	165 ± 15^a^	94.4 ± 8.4^c^	117 ± 14^b^	175 ± 9.2^a^
Epididymal fat pads/body weight (g/kg body weight)	11.3 ± 1.0^a^	11.0 ± 0.7^a^	9.9 ± 0.5^b^	10.1 ± 0.8^b^	11.0 ± 1.0^a^
Retroperitoneal fat/body weight (g/kg body weight)	11.1 ± 0.7^a^	11.3 ± 0.9^a^	9.8 ± 0.9^b^	9.2 ± 0.7^b^	11.1 ± 1.0^a^
Visceral fat (g/kg body weight)	22.4 ± 1.6^a^	22.3 ± 1.4^a^	19.7 ± 1.1^b^	19.3 ± 1.7^b^	22.1 ± 1.9^a^
Food intake (g/day)	16.6 ± 1.8^a^	9.7 ± 1.0^b^	10.3 ± 2.1^b^	15.8 ± 1.7^a^	16.7 ± 1.6^a^
Energy intake (kcal/day)	71.3 ± 7.7a	63.8 ± 6.6b	44.4 ± 9.0c	61.5 ± 6.6b	71.6 ± 6.9a
Energy expenditure (kcal/body weight/day)	103 ± 10^ab^	110 ± 11^a^	117 ± 12^a^	110 ± 11^a^	94 ± 11^b^
Respiratory quotient	0.86 ± 0.09^ab^	0.81 ± 0.09^b^	0.82 ± 0.09^b^	0.92 ± 0.08^a^	0.81 ± 0.08^b^
Carbohydrate oxidation during dark cycle (mg/kg body weight/min)	5.8 ± 0.7^b^	4.1 ± 0.7^c^	4.7 ± 0.7^c^	8.7 ± 0.9^a^	3.5 ± 0.5^d^
Fat oxidation during dark cycle (mg/kg body weight/min)	5.2 ± 0.6^c^	7.6 ± 0.8^a^	7.8 ± 0.9^a^	3.0 ± 0.4^d^	6.5 ± 0.8^b^

The amyloid-β(25–35) infused diabetic rats AD model rats received assigned diet for 8 weeks as follows: 1) a normal diet (28 energy % fat diet) with *ad libitum* feeding (AD-CON), 2) ketogenic diet (AD-KD; 0.1% carbohydrate diet), 3) high carbohydrate diet (AD-CHO; 68 energy % carbohydrate diet) and 4) normal diet with intermittent fasting (AD-IMF). The sham-operated rats received the same diet (normal diet) as AD-CON (Non-AD-CON) for 8 weeks. Values represent means ± SD. ^a,b,c,d^Different superscripts after SD indicate significant differences at *p*<0.05.

**Table 3:** Lipid profiles and glucose and insulin levels in the circulation of overnight-fasted rats

	AD-CON (*n* = 10)	AD-KD (*n* = 10)	AD-IMF (*n* = 10)	AD-CHO (*n* = 10)	Non-AD-CON (*n* = 10)
Serum β-hydroxy butyrate (mM)	0.15 ± 0.05^c^	0.40 ± 0.09^a^	0.24 ± 0.07^b^	0.14 ± 0.07^c^	0.15 ± 0.06^c^
Fasting serum glucose (mg/dl)	126.3 ± 11.4	128.0 ± 11.4	122.2 ± 15.4	119.8 ± 12.9	120.1 ± 12.1
Fasting serum insulin (ng/ml)	2.52 ± 0.39^b^	2.51 ± 0.28^b^	3.01 ± 0.41^a^	1.86 ± 0.26^c^	2.01 ± 0.32^c^
HOMA-IR	14.1 ± 1.5^b^	14.3 ± 1.3^b^	16.6 ± 2.1^a^	9.9 ± 1.2^c^	10.7 ± 1.3^c^
Total cholesterol (mg/dl)	83.9 ± 9.1^b^	73.2 ± 5.9^c^	91.7 ± 9.7^b^	108 ± 10^a^	75.4 ± 8.5^bc^
HDL cholesterol (mg/dl)	23.6 ± 3.2^b^	30.2 ± 3.8^a^	21.9 ± 3.2^b^	17.6 ± 2.7^c^	24.1 ± 3.1^b^
LDL cholesterol (mg/dl)	47.7 ± 5.5^c^	33.2 ± 3.9^e^	60.5 ± 6.2^b^	77.8 ± 8.2^a^	40.9 ± 4.9^d^
Triglyceride levels (mg/dl)	62.7 ± 5.8^a^	54.4 ± 6.3^b^	60.2 ± 5.6^a^	63.7 ± 6.3^a^	53.2 ± 6.3^b^

The amyloid-β(25–35) infused diabetic rats AD model rats received assigned diet for 8 weeks as follows: 1) a normal diet (28 energy % fat diet) with *ad libitum* feeding (AD-CON), 2) ketogenic diet (AD-KD; 0.1% carbohydrate diet), 3) high carbohydrate diet (AD-CHO; 68 energy % carbohydrate diet) and 4) normal diet with intermittent fasting (AD-IMF). The sham-operated rats received the same diet (normal diet) as AD-CON (Non-AD-CON) for 8 weeks. Values represent means ± SD. ^a,b,c,d^Different superscripts after SD indicate significant differences at *p*<0.05.
